# Functional Brain Network Modularity Captures Inter- and Intra-Individual Variation in Working Memory Capacity

**DOI:** 10.1371/journal.pone.0030468

**Published:** 2012-01-20

**Authors:** Alexander A. Stevens, Sarah C. Tappon, Arun Garg, Damien A. Fair

**Affiliations:** 1 Department of Psychiatry, Oregon Health & Science University, Portland, Oregon, United States of America; 2 Department of Behavioral Neuroscience, Oregon Health & Science University, Portland, Oregon, United States of America; University of British Columbia, Canada

## Abstract

**Background:**

Cognitive abilities, such as working memory, differ among people; however, individuals also vary in their own day-to-day cognitive performance. One potential source of cognitive variability may be fluctuations in the functional organization of neural systems. The degree to which the organization of these functional networks is optimized may relate to the effective cognitive functioning of the individual. Here we specifically examine how changes in the organization of large-scale networks measured via resting state functional connectivity MRI and graph theory track changes in working memory capacity.

**Methodology/Principal Findings:**

Twenty-two participants performed a test of working memory capacity and then underwent resting-state fMRI. Seventeen subjects repeated the protocol three weeks later. We applied graph theoretic techniques to measure network organization on 34 brain regions of interest (ROI). Network modularity, which measures the level of integration and segregation across sub-networks, and small-worldness, which measures global network connection efficiency, both predicted individual differences in memory capacity; however, only modularity predicted intra-individual variation across the two sessions. Partial correlations controlling for the component of working memory that was stable across sessions revealed that modularity was almost entirely associated with the variability of working memory at each session. Analyses of specific sub-networks and individual circuits were unable to consistently account for working memory capacity variability.

**Conclusions/Significance:**

The results suggest that the intrinsic functional organization of an *a priori* defined cognitive control network measured at rest provides substantial information about actual cognitive performance. The association of network modularity to the variability in an individual's working memory capacity suggests that the organization of this network into high connectivity within modules and sparse connections between modules may reflect effective signaling across brain regions, perhaps through the modulation of signal or the suppression of the propagation of noise.

## Introduction

Common experience reminds us that on some days we do not feel as mentally astute as on others. A poor night's sleep, illness, or stressful life events all may influence our cognitive faculties and lead to variability in performance on different days. This intra-individual variability stands in contrast to the well-documented individual differences in intellectual performance.

Working memory, as measured by visual short-term memory (VSTM) capacity, is typically considered a stable characteristic of an individual's cognitive functions and is closely linked to individual differences in human intellectual abilities [1]–[3]. However, endogenous and exogenous factors such as sleep disruption [4], psychosocial stress [5], and pharmacological manipulations [6] create intra-individual variability in cognitive performance, such that an individual's working memory capacity may vary at different times. Such systemic, psychological, and environmental factors influence daily cognitive variability via multiple brain network interactions. These factors are also likely reflected in the organization of brain systems necessary to carry out complex cognitive functions [7]–[9]. Working memory capacity, as measured using visual short term memory (VSTM) tasks is dependent on specific neural systems to maintain representations, with posterior parietal areas being particularly important [10], [11]. The observed working memory capacity of an individual also depends on the organization and interaction of multiple brain regions that are commonly observed to be engaged in numerous cognitive functions [12]–[14]. Thus, true working memory capacity may be stable across time, but expressed capacity may vary as a result of the changes in cognitive control systems. This would explain why cognitive performance across different domains tends to vary together. Measures of functional organization of control systems may capture important sources of variability both between and within individuals across time.

We hypothesized that the functional organization of a system of brain regions implicated in a broad range of cognitive functions may predict working memory capacity in terms of individual differences. Furthermore, when measured on different days, the organization of these networks may also predict intra-individual variability in working memory capacity. The coherent organization of these networks during the absence of externally directed tasks (i.e., “resting state”) may reflect the latent cognitive abilities of the brain at that time. Indeed, previous studies have demonstrated that low frequency variation in functional connectivity between brain regions, measured in the absence of experimenter imposed tasks, is associated with the functional roles attributed to those brain regions [15], [16]. Graph-theoretic tools provide quantitative measurements of complex patterns of organization across a network, and have been effective in informing brain-behavior relationships based on functional and structural connectivity [17]–[20]. Recently, graph-theoretic analysis has been applied to human functional brain connectivity and specifically to detection and characterization of community structure in networks [21]–[24]. For example, small-world measures calculated on resting-state functional connectivity have revealed that network efficiency predicted individual differences in intellectual ability [25]. These findings suggest that the quantitative measures of network organization at a global network level capture important information about brain and behavior relationships, even when measured in the absence of experimenter-imposed tasks [26]. Here, we tested the hypothesis that graph-theoretic measures of a functional brain network vary across days, and that this variation is linked to variation in visual short-term memory capacity both between and within individuals across time.

We examined 34 brain regions (**Table 1**) that have been empirically identified in previous neuroimaging studies across a range of cognitive tasks [8], [23], [27], [28]. We were particularly interested in the organizational properties of the network as a whole rather than specific connections or sub-networks. Therefore, we focused on network modularity, a statistic reflecting how well an entire network is organized into modules of densely interconnected nodes, but with the modules only sparsely interconnected [29]–[31]. Modularity captures an important organizational principle critical to biological systems; integration within sub-systems allows efficient local processing, while sparse connections between sub-systems reduce the propagation of noise [32]. We specifically hypothesized that since greater modularity characterizes optimal system organization, then the modularity of a network of brain regions associated with broad cognitive mechanisms should relate to cognitive performance, measured here as working memory capacity. Similarly, we expected another measure of network efficiency, small-worldness, to be associated with VSTM capacity. Small-worldness provides a somewhat different measure of organization of networks, reflecting high local inter-connectivity coupled with a shorter than expected distances between any two nodes due to sufficient inter-module connectivity or the presence of “hubs,” nodes that are densely connected to other nodes [33].

**Table 1:** Regions of interest used as nodes in the network network analysis, drawn from [22], [23].

		Coordinates			
Whole-brain region of interest (ROIs)	Abbreviation	X	Y	Z	Module
dorsoloateral prefrontal cortex	L.dlPFC	−43	22	34	Fonto-parietal
dorsoloateral prefrontal cortex	R.dlPFC	43	22	34	Fonto-parietal
Frontal	L.frontal	−41	3	36	Fonto-parietal
Frontal	R.frontal	41	3	36	Fonto-parietal
mid cingulate cortex	mCC	0	−29	30	Fonto-parietal
inferior parietal lobule	L.IPL	−51	−51	36	Fonto-parietal
inferior parietal lobule	R.IPL	51	−47	42	Fonto-parietal
intraparietal sulcus	L.IPS	−31	−59	42	Fonto-parietal
intraparietal sulcus	R.IPS	30	−61	39	Fonto-parietal
Precuneus	L.precuneus	−9	−72	37	Fonto-parietal
Precuneus	R.precuneus	10	−69	39	Fonto-parietal
anterior Prefrontal cortex	L.aPFC	−28	51	15	Cingulo-Opercular
anterior Prefrontal cortex	R.aPFC	27	50	23	Cingulo-Opercular
anterior insula/frontal operculum	L.aI/fO	−35	14	5	Cingulo-Opercular
anterior insula/frontal operculum	R.aI/fO	36	16	4	Cingulo-Opercular
dorsal anterior cingulate/medial superior frontal cortex	dACC/msFC	−1	10	46	Cingulo-Opercular
superior frontal cortex	L.ant.thalamus	−12	−15	7	Cingulo-Opercular
anterior thalamus	R.ant.thalamus	10	−15	8	Cingulo-Opercular
anterior thalamus	amPFC	1	54	21	Cingulo-Opercular
ventromedial prefrontal cortex	vmPFC	−3	39	−2	Default
superior frontal cortex	L.sup.frontal	−14	38	52	Default
superior frontal cortex	R.sup.frontal	17	37	52	Default
inferior temporal	L.inf.temporal	−61	−33	−15	Default
inferior temporal	R.inf.temporal	65	−17	−15	Default
parahippocampal	L.parahippocampal	−22	−26	−16	Default
parahippocampal	R.parahippocampal	25	−26	−14	Default
posterior cingulate cortex	pCC	−2	−36	37	Default
lateral parietal	L.lat.parietal	−47	−67	36	Default
lateral parietal	R.lat.parietal	53	−67	36	Default
retrosplenial	retrosplenial	3	−51	8	Cerebellar
lateral cerebellum	L.lat.cerebellum	−32	−66	−29	Cerebellar
lateral cerebellum	R.lat.cerebellum	31	−61	−29	Cerebellar
inferior cerebellum	L.inf.cerebellum	−19	−78	−33	Cerebellar
inferior cerebellum	R.inf.cerebellum	18	−80	−33	Cerebellar

## Results

**Figure 1:**
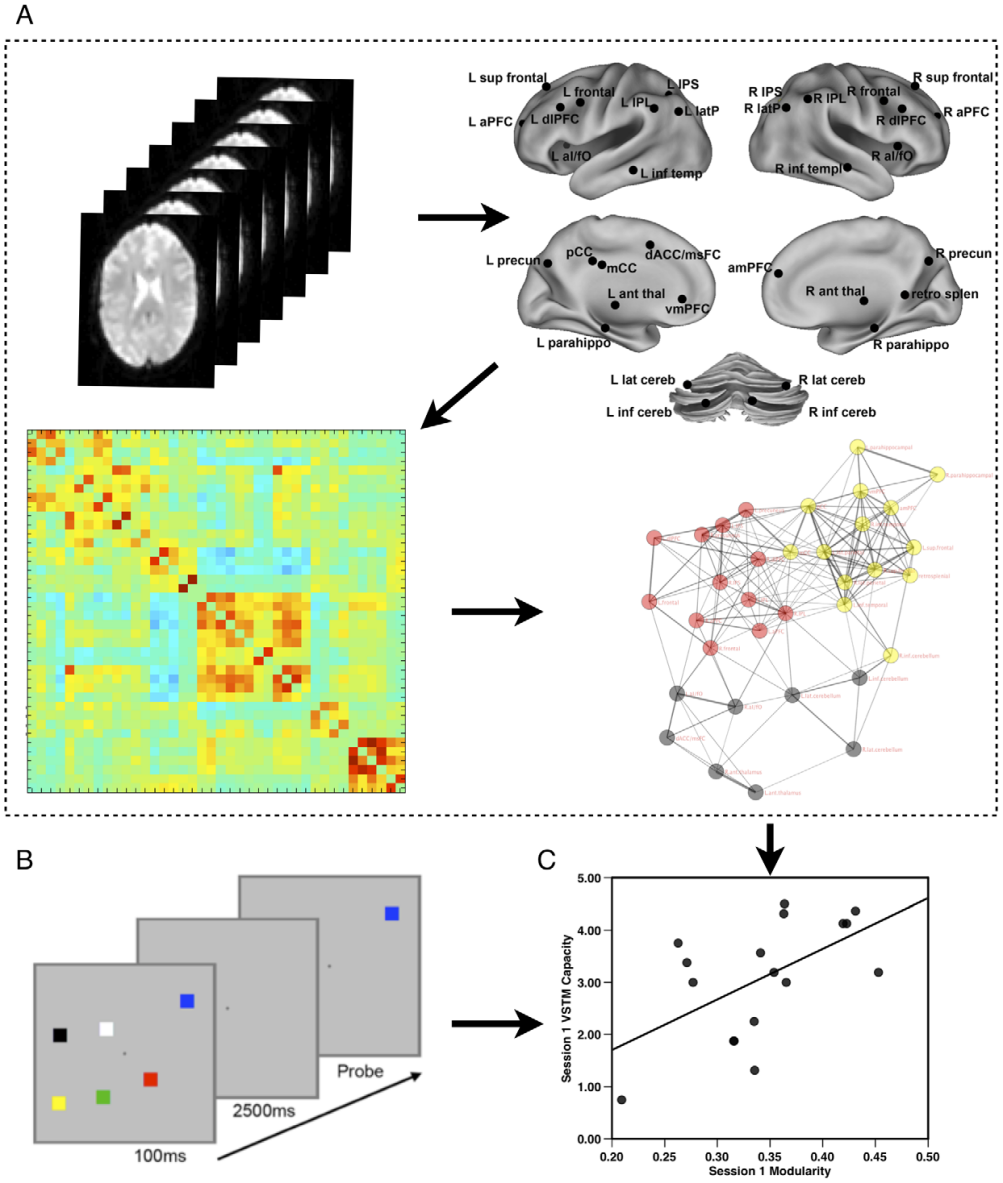
Study design for a single session. The visual short-term memory task was administered prior to the resting state scans in Session 1 and three weeks later at Session 2. **a**) Functional MRI was conducted while subject maintained relaxed fixation on a crosshair. Low-frequency time course data was extracted from 34 brain regions (nodes) for each subject and a correlation matrix of all 34 nodes was constructed and binarized at a threshold of r = 0.25 for each participant. The community structure (modularity) of each participant's network was determined using a modularity maximization technique [30], and is here visualized using a spring-embedded graph, with node colors indicating module membership (see Supplement for details). **b**) Example of the six-object VSTM task used to estimate working memory capacity. The individual participant's capacity estimate and network modularity measures were used to compute **c**) the correlation at each session and changes between sessions.

Twenty- two participants completed an initial session and 17 returned three weeks later for a second identical session. At each session, they performed a VSTM task to provide memory capacity estimates (**Fig. 1B**) followed by resting-state fMRI scans to measure the functional connectivity among 34 regions (**Fig. 1A** **,** **Table 1**). Network statistics presented here were calculated on each participant's binarized graphs with a correlation threshold of r = 0.25. This threshold was based on several criteria (**Text S1**); however, the results hold over multiple thresholds (**Fig. S1**).

**Figure 2:**
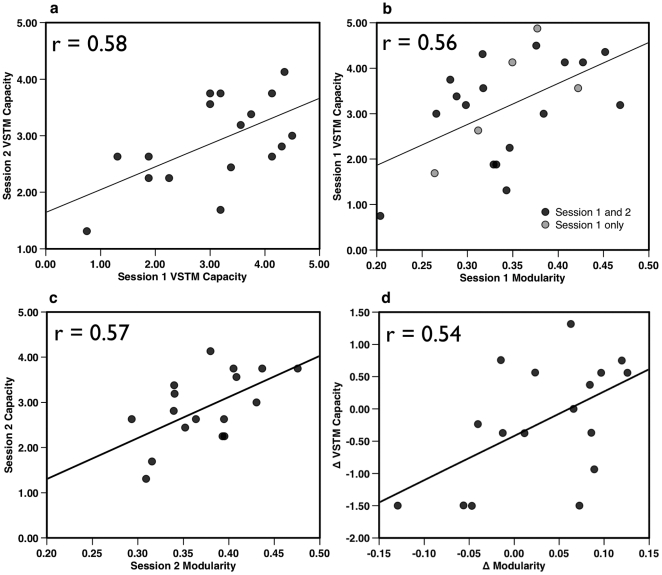
Association of Modularity and Visual short-term memory (VSTM) capacity. VSTM capacity measures were reliably correlated across **a**) Sessions 1 and 2 (p = 0.014). **b**) Modularity and VSTM capacity measured in Session 1 (n = 22) were correlated (p = 0.009) as well as **c**) when measured three weeks later at Session 2 (p = 0.017, n = 17). **d**) Individual participant's changes in modularity and VSTM capacity between Session 1 and Session 2 were also strongly correlated (p = 0.014), indicating that changes in subject's VSTM capacity were reflected in the change in their network modularity. All tests two-tailed.

**Figure 3:**
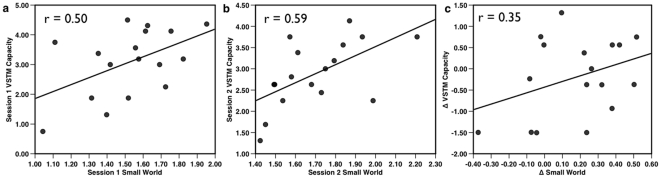
Small-worldness correlated with VSTM capacity. At: a) Session 1 (p = 0.042), and b) Session 2 (p = 0.013). c) Changes in small-worldness between Session 1 and Session 2 were not significantly correlated with change scores of VSTM capacity (p = 0.17). All tests two-tailed.

Individual modularity values ranged over 0.21–0.49, while the within-subject changes in modularity ranged from 0.01–0.14. Thus, modularity within individuals tended to be relatively stable compared to the differences between individuals. Visual short-term memory capacity was comparable across Session 1 (mean = 3.09) and Session 2 (mean = 2.89): paired t(16) = 0.834, p = 0.34. Individual's network modularity and VSTM capacity were positively correlated at both Session 1 (r(20) = 0.56, p = 0.009) (**Fig. 2a**), and Session 2 (r(15) = 0.57, p = 0.008), indicating that working memory capacity indeed varies reliably with network modularity. (When only the 17 participants who completed both Sessions 1 and 2 were included in the analysis, the correlation between network modularity and VSTM at Session 1was comparable: r(15) = 0.54, p = 0.013). Strikingly, within-individual changes in VSTM capacity between Session 1 and Session 2 also correlated with the change in their network modularity between the two sessions (r(15) = 0.54, p = 0.013). Thus, network modularity not only captured significant variability in individual differences in working memory capacity, but also tracked the within-individual changes in memory between sessions. Importantly, another measure of global network organization, small-worldness (**Fig. 3**), yielded similar correlations with VSTM capacity at both Session 1 (r (20) = 0.50, p = 0.042) and Session 2 (r(15) = 0.59, p = 0.013). However, unlike modularity, between-session changes in small-worldness were only weakly associated with changes in VSTM capacity (r(15) = 0.35, p = 0.17) (**Fig. 3**). Thus, like modularity, small-worldness is sensitive to individual differences in VSTM capacity; however, unlike modularity, it is relatively insensitive to intra-individual variability in capacity.

### Modularity is associated with working memory variability

Based on the finding that changes in VSTM capacity between sessions correlate with changes in network modularity, we further reasoned that modularity might be most closely linked to the variable component of VSTM, rather than the stable component. The covariation in working memory capacity estimates at Sessions 1 and 2 (**Fig. 2a**) provides the best estimate of an individual's stable working memory capacity, by reducing the influence of measurement error and other sources of fluctuation that contribute to variability in test scores. This stable component is reflected in the correlation between capacity measured in Session 1 with that measured in Session 2. We reasoned that the residual variance in capacity estimates across the two sessions, rather than reflecting simply measurement error, may contain additional information regarding day-to-day variation in VSTM capacity due to the effectiveness of the organization of the underlying neural systems involved in cognitive performance. We demonstrated that this was indeed the case using partial correlations. Specifically, we removed the variance in VSTM capacity in one session attributable to VSTM capacity measured in the other session (stable VSTM capacity) and determined if network modularity accounted for the remaining variance in VSTM capacity. Partial correlation between Session 1 VSTM capacity and modularity, controlling for Session 2 capacity, remained reliable (partial r(14) = 0.50, p = 0.046, two-tailed). A similar relationship also held for modularity and capacity at Session 2 with Session 1 capacity removed (partial r(14) = 0.51, p = 0.045, two-tailed). In both cases the partial correlations were comparable to their respective zero-order correlations between same-session modularity and capacity (**Fig. 2**). Modularity accounted for 25% of the variability in VSTM capacity measured on the same day over and above the variance accounted for by the same individual's capacity measured at the other testing session. This indicates that network modularity was indeed linked more closely to *variability* in VSTM capacity rather than to the working memory capacity component that was stable across sessions. Together the stable component of working memory capacity (the correlation between working memory capacity sessions 1 and 2) and the variable component in working memory capacity that was accounted for by modularity in sessions 1 and 2, accounted for over 50% of participants' working memory capacity at each session.

### Working memory is not associated with sub-network components

**Figure 4:**
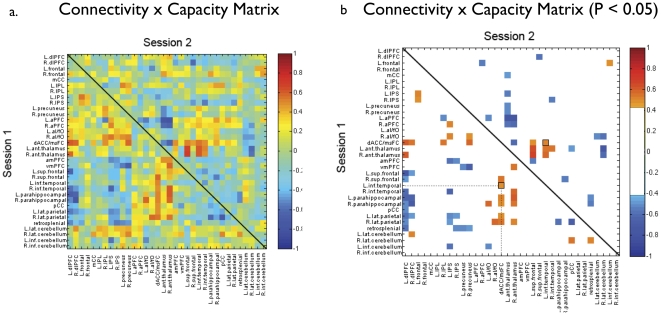
The correlation matrix for connection weights between nodes and VSTM. Capacity **a**) in Session 1 (lower triangle) and Session 2 (upper triangle). **b**) For clarity, the same correlation matrix with non-significant connections eliminated (threshold r = 0.47, p≤0.05, uncorrected for multiple correlations).

In order to ensure that the observed association between network level measures and inter- and intra-individual differences in working memory capacity were not due to simple connections between pairs of nodes or other sub-network information, we examined the relationship between all possible node pairs and VSTM capacity at both sessions. Only one pair of nodes (right parahippocampus – right thalamus) was correlated with capacity at both sessions (uncorrected for multiple correlations) (**Fig. 4**). Based on an uncorrected probability of p≤0.05 at Session 1 and Session 2, we would expect 1.4 node pairs to be significantly correlated with VSTM capacity at both sessions by chance. The specific organization of the nodes within the modules of the network did not appear to be critical to working memory capacity. For instance, connection density within the modules of the network, which were defined in previous reports [22], [23], may have accounted for the observed relationship between modularity and working memory. Therefore, we determined the connection density based on the binarized and thresholded networks, and summed the weights of connections within the sub-networks. However, for none of the individual modules did the degree of global intra-modular connectivity reliably account for working memory capacity. Similarly, the connectivity strength between modules of these previously defined nodes failed to produce significant correlation with working memory capacity (**Table 2**). These results suggest that the organization of the nodes into specific modules is not critical, and that there may be many ways in which these neural systems can create effective functional organizations.

**Table 2:** Correlations between VSTM capacity and intra-module connection density, and between module connectivity.

Network modules	Session 1	Session 2	Change
Fronto-Parietal	0.28	0.09	−0.12
Cingulate-Operculum	−0.06	−0.58*	−0.35
Default Mode Network	−0.31	−0.41	0.13
Within-module connectivity across network	−0.17	−0.78**	−0.33
Between Module Connectivity	−0.35	−0.11	−0.25

*P<0.05, uncorrected;

**P<0.01, uncorrected.

**Figure 5:**
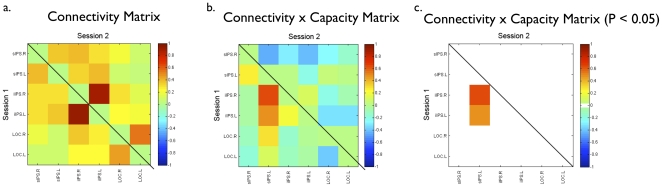
Connectivity matrix for the six regions of interest from Xu & Chun (2006). The matrices indicate a) the intercorrelations among node-pairs for Session 1 (lower triangle) and Session 2 (upper triangle), b) the correlation between VSTM capacity and the strength of correlation for each node pair at Session 1 and Session 2, and c) the same matrix as in b but set to a threshold of r = 0.47, p = 0.05, uncorrected for multiple comparisons. Abbreviations of anatomical locations: sIPS.R - Right superior intra-parietal sulcus, sIPS.L - Left superior intra-parietal sulcus, iIPS.R - Right inferior intra-parietal sulcus, iIPS.L - Left inferior intra-parietal sulcus, LOC.R - Right lateral occipital cortex, LOC.L - Left lateral occipital cortex.

One logical alternative network that could account for working memory capacity is the set of regions reported to be active during a VSTM task [34], [35]. Therefore we used as nodes, the six regions of interest specifically related to VSTM capacity identified in the study reported by Xu and Chun [34], which subsumed the regions reported by Todd and Marois [35]. Because the set of six regions was too small to submit to meaningful graph analysis, we calculated the correlation matrix among all six nodes and determined if any functional connections consistently predicted VSTM capacity (**Fig. 5**). No consistent correlation between VSTM capacity and connectivity between node-pairs was evident, indicating that resting state connectivity is likely tapping into brain interactions that are different from those detected during task-related activity. This result may reflect the possibility that separate mechanisms important for information processing may be detected by these different functional imaging approaches. For instance, resting-state may be capturing general functional organization of the brain (the cognitive “tone” of the brain), while event-related fMRI captures the actual responsiveness of these brain regions during task-based activity.

## Discussion

The current study used network theoretic measures applied to resting state fMRI data to determine if the organization of a specific network of generalized control regions was associated with variability in working memory capacity. Modularity, and to a similar degree, small-worldness of this network, reliably accounted for individual differences in working memory capacity at both session 1 and session 2. The replication of this relationship at two separate sessions provided a robust test of the reliability of the relationship between network organization and working memory. More strikingly, the change in an individual's network modularity between the two sessions predicted the change in working memory capacity. These observations together suggest that organizational characteristics of a broad functional network across a variety of cognitive tasks contain information about latent cognitive functions [8].

We specifically focused on these global network variables due to mounting evidence that organizational properties of an entire functional network are linked to intellectual functions and learning [25], [26], and therefore may capture properties of effective cognitive functioning. Working memory, which contributes to many aspects of daily cognition, is likely to be a particularly good bell-weather for variation in cognitive performance. It is important to stress that we do not interpret the relationship between modularity and working memory capacity to indicate that the defined network is responsible for working memory *per se.* Rather, we interpret these results to reflect brain-behavioral relationships that are likely to extend across a variety of cognitive demands. This interpretation is consistent with the set of ROIs used here, which were selected based on their consistent activity across a wide range of cognitive tasks and studies [22], [23], [37], [38]. It is possible that systemic factors, life stressors, and sleep quality, which all have demonstrable effects on cognitive functions, including working memory [4]–[6], may leave their imprint on the functional organization of neural systems associated with a broad array of generalized control related functions.

One issue that remains only partially resolved in this study is whether specific circuits among the nodes accounts for working memory capacity equally well or better than their global organization as measured via modularity. While we did not exhaust the full extent of possibilities, we did attempt to rule out some alternative explanations of this sort. For example, we did not find a reliable association with working memory capacity at the level of single nodal connections (**Figure 5**), nor at the level of sub-networks (**Table 2**). This included an examination of fronto-parietal systems that are clearly involved in visuo-spatial working memory functions [12], [13], [36]. In the same vein, we tested a set of posterior parietal regions identified in previous event-related fMRI studies of working memory capacity that employed very similar VSTM tasks to the task used in the current study to measure working memory capacity [11], [34], [35]. These studies converged on a small set of posterior parietal regions that were very closely linked to working memory capacity. However, the analysis of resting-state connectivity among this small set of nodes [11], [35] also failed to detect an association with working memory capacity. These results do not rule out the possibility that there is an optimal set of nodes whose organization predicts working memory from resting state data, nor can we be sure that other brain regions not included in the network may provide important predictive information about latent cognitive functions. However, brain regions included as nodes in this study have been repeatedly implicated in a wide range of control related functions identified during fMRI studies where active tasks were compared to resting baseline conditions [8], [37]–[39]. Therefore, the information contained in resting state networks appears to complement evidence from event-related fMRI studies [11], [35] and electrophysiological evoked potential data [10].

The current work does not attempt to identify whether there is a single optimal brain organization that determines cognitive performance. However, there is some evidence that while performance appears to be determined by the efficient neural interactions among brain systems, multiple “optimal” configurations may exist. For example, a recent fMRI study by Bassett and colleagues, reported changes in functional connectivity while individuals acquired a complex motor task [26]. They found that while modularity of the group was relatively stable across time, a measure of “flexibility”, described as the degree to which individual nodes would shift between modules, was predictive of subsequent changes in motor learning [26]. Bassett and colleagues did not report modularity as a predictor of individual performance, but their results suggest that a specific organization may not be a critical starting point as long as modularity is reasonably high.

A limitation to this study, common to most “resting-state” investigations, is that the experimenter gives up control over the structure of the stimulation of the brain. Consequently, it is possible that the correlation we observed may reflect differences in the internal thought-processes of the individuals, or other unconstrained variable, which may be directly or indirectly related to working memory capacity. However, such a variable must also be linked to the modularity and small-world characteristics of the functional network.

Performance of complex biological systems has been theorized to depend on a modular organization in which there are highly integrated subsystems that are largely segregated from each other [17], [18], [32]. While the integration within a module seems clearly necessary for information to be shared between relevant processing elements, the segregation between modules may be equally necessary to avoid proliferating internal or external noise that could potentially interfere with task-relevant functions. Multiple sources of noise can interfere with cognitive processes and modularity of a network attenuates the propagation of noise between modules [31]. This may explain why working memory was specifically tied to the global community structure of the network, but not to individual nodes or connections between nodes. Future work combining task-based fMRI and resting state fMRI analyses conducted in the same studies, may further clarify how the unique brain signals measured with these different imaging techniques account for different sources of variation in cognitive performance. It may be that efficient processing within these regions combined with the interactions between them (even when unchallenged by an externally imposed task), reflect the state of organization of neural systems necessary for complex cognitive tasks [43].

## Materials and Methods

### Ethics Statement: This research protocol was reviewed and approved by the OHSU Research Integrity Office's Internal Review Board

#### Participants

In the initial experimental session, 22 individuals (15 females, 7 males; mean age = 27.5 yrs; SD = 4.8 yrs) participated in the first session after providing written informed consent. Seventeen of the original participants returned for an identical session three weeks after their first visit. All participants were right-handed, had normal or corrected-to-normal vision, and had no history of brain damage, learning disability, psychiatric diagnosis, or neurological problems. Informed written consent was obtained from all participants.

#### Visual Short-Term Memory task

The visual working memory procedure was based on the task described by Vogel and colleagues [40]. It required participants to remember two or six colored squares presented briefly (100 ms) on a computer monitor. Following a 2500 ms delay, a probe stimulus was displayed and participants indicated whether it was the same color and location as one of the squares from the original stimulus set. In a third of the trials, the two colored squares were presented with four distracters in the form of colored rectangles. Participants were told to ignore the distracters and only remember the squares [40]. Working memory capacity estimates were computed for the six-stimulus condition, as capacity K = s*(H - F), where *s* is the number of stimuli in the memory array, H is the number of hits and F is the number of false alarms [41]. At each session, participants completed 192 trials, 64 of each condition inter-mixed in four blocks of 48 trials.

#### MRI data acquisition

All MRI scans were performed on a Siemens 3 Tesla TIM-TRIO system. Structural images were obtained using a sagittal magnetization-prepared rapid gradient echo (MP-RAGE) three-dimensional T1-weighted sequence (TR = 9.7 ms, TE = 4 ms, flip angle = 12°, TI = 300 ms, voxel size = 1.25×1×1 mm, slices = 128). Functional images were obtained using a gradient-echo, echo-planar sequence sensitive to blood oxygen level-dependent (BOLD) contrast (TR = 2000 ms; TE = 30 ms; FOV = 240 mm^3^; flip angle = 90°). Participants completed two scans consisting of 150 acquisitions of 33 contiguous interleaved 3.8 mm axial slices, which were acquired parallel to the plane transecting the anterior and posterior commissure. This covered the entire cerebrum and all but the most inferior portion of the cerebellum. Steady state magnetization was assumed after four frames (∼8 seconds).

#### Functional MRI Data preprocessing

The resting state fMRI preprocessing included (i) removal of a central spike caused by MR signal offset, (ii) correction of odd vs. even slice intensity differences attributable to interleaved acquisition without gaps, (iii) correction for head movement within and across runs, and (iv) within-run intensity normalization to a whole brain mode value gradient of 1000. Atlas transformation^3^ of the functional data was computed for each individual via the MP-RAGE. Each rs-fMRI run was then resampled in atlas space on an isotropic 3.0 mm**^3^** grid combining the six parameters for movement correction and atlas transformation in one interpolation. All subsequent operations were performed on the atlas-transformed volumetric time series. As previously described [4], several additional preprocessing steps were used to reduce spurious variance unlikely to reflect neuronal activity. These steps included: (a) a temporal band-pass filter (0.009 Hz<f<0.08 Hz) and spatial smoothing (6 mm full width at half maximum), (b) regression of six parameters obtained by rigid body head motion correction, (c) regression of the whole brain signal averaged over the whole brain, (d) regression of ventricular signal averaged from ventricular ROI, and (e) regression of white matter signal averaged from white matter ROI. Regression of first order derivative terms for the whole brain, ventricular, and white matter signals were also included in the correlation preprocessing.

Motion-related signal was also removed from all analyses between graph theoretic measures and VSTM capacity. For each subject, the root mean square of the sum of the estimates of the six orthogonal motion parameters was calculated for each fMRI scan and averaged within-session. This was then regressed against the statistic of interest (e.g., Modularity) and the residual of the regression used as the corrected estimate of the statistic. The residuals were re-expressed in original units around the mean value of the statistic (e.g., corrected Modularity).

#### Regions of interest

The ROIs (**Table 1**) were selected based on earlier studies of cortical regions that were detected across multiple studies using a variety of cognitive tasks. The ROIs included nodes identified as part of the default-mode network [27], [28]. Each ROI (node) consisted of a 10 mm^3^ sphere centered on the peak loci of activation reported in those studies. Mean time courses were extracted from the 34 ROIs in previous work. Each participant's ROI timecourses were inter-correlated to form a 34×34 connectivity matrix representing the strength of connection between every pair of ROIs. Subsequent network analyses were performed on these matrices.

### Graph Construction

All network measures were calculated using functions written in the Brain Connectivity Toolbox created by Sporns and colleagues (https://sites.google.com/a/brain-connectivity-toolbox.net/bct/Home) The formulae for the network measures are provided in their recent paper [8]. For each individual dataset, the connectivity graph was constructed by creating a 34×34 correlation matrix M. We then created a binarized matrix (B) formed by thresholding each M by a threshold T. All values of cells of the correlation matrix<T were then set to equal 0. All other cells were set equal to 1. Binarizing a graph requires choosing a threshold value of r above which a connection between two nodes is considered to exist. Setting a threshold too low carries a risk of over-estimating the influence of noisy, physiologically insignificant weak correlations, thereby losing sensitivity to the underlying network organization. Conversely, very high thresholds can cause excessive fragmentation and may misrepresent the graph structure by disregarding correlations of potential functional importance. As no definitive method of selecting an optimal threshold is currently available, we performed our analyses at a range of plausible thresholds, but reported results using a threshold for visualizations (r = 0.25) based on the characteristics described below. This threshold was determined by satisfying the following criteria (also see **Figure S1**). First, the network must differ from a random graph on measures of modularity and small-world properties. Random graphs were constructed using randomly placed connections constrained to have the same degree distribution as the real graphs [9]. This comparison across different binarization thresholds indicates at what threshold values, the apparent structure of the graph is unlikely to have resulted by chance. Additionally, the average reachability of the network, the probability that a direct or indirect path exists between any two nodes in the graph, must not differ significantly from 1.0. Setting T to correlations of 0.25 to 0.30 satisfied these conditions for all individual subject connectivity maps. We note that this approach allows the number of connections included in the network to vary across participants.

We also performed the network thresholding using the connection density thresholds to binarize the individual participants correlation matrices (**Text S1**). Unlike correlation based thresholds which allow the number of connections in the network to vary between subjects, connection density holds the number of connections in the network constant by retaining the specified proportion of connections determined by correlation strength. While this approach produced similar results at session 1, in terms of the relationship between small-worldness and VSTM capacity, it failed to reveal the same relationship at Session 2 (**see Figure S2**).

#### Reachability

A measure of the proportion of nodes that are connected by any path, regardless of the path length (i.e. the number of intervening nodes that must be traversed in order to reach one node from the other). A low reachability score implies a fragmented graph where some nodes are completely disconnected from all others in the network, which is biologically implausible as a model of the brain [8].

#### Small-worldness

A measure of global network organization, reflects high local inter-connectivity coupled with a shorter than expected distances between any two nodes due to the presence of “hubs” - nodes that are densely connected to other nodes. It is defined in terms of clustering coefficient and characteristic path length [10], [11]. Characteristic path length (λ) is a measure of the mean number of links that must be traversed in order to travel between any two nodes in the network. Clustering coefficient (C) is a measure of the average proportion of a node's nearest neighbors that are also connected to each other. Small-world networks possess a clustering coefficient that is much larger than a random graph, and a characteristic path length that is equal or only slightly larger than in a random graph. The ‘small-worldness’ of a network (S) can thus be quantified as (C_real_/C_random_)/(λ_real_/λ_random_) [11], [12].

#### Modularity estimation

Modularity, or community structure, is a technique for subdividing the network into separate modules (communities), which maximize the connections within each module and minimize the connections between modules [8], [9], [13]. Modularity is estimated using optimization algorithms. Network modularity values in the current study were estimated using the method of Newman [9] and then confirmed using the algorithm developed by Blondel [13]. As the Blondel modularity algorithm is non-deterministic and can yield slightly different values of modularity for a given network across multiple runs, we calculated modularity using both these techniques for each subject by averaging across 100 iterations of the algorithm. The two different modularity estimates converged to near unity at both sessions (Session 1: r(15) = 0.98, p<0.001; Session 2: r(15) = 0.98, p<0.001). We further tested the reliability of the modularity values using the Variation of Information, a measure of the stability of the network's organization [42] (**Text S1, Figure S3**).

## Supporting Information

Figure S1 **Graphs demonstrating the divergence of the real graphs from random graphs as a function of matrix thresholds for different measures of network organization.** The individual networks obtained in Session 1 and Session 2 were compared to random networks constructed with the same number of nodes, connections and connection density. **a**) Reachability did not differ significantly from 100% when thresholds correlations were set below r = 0.30 (maximum *t(16)* = −1.5, *p* = .16), indicating that these thresholds preserve full connectedness of the network in most individual networks. **b**) Characteristic path length, and c) clustering coefficient were significantly different from the random graphs across all threshold correlations (r), where 0.05≤r≤0.50. **c**) Small-worldness (S), calculated as a ratio (Clustering_real_/Clustering_random_)/(Path Length_real_/Path Length_random_), such that values different from 1.0 reflect a departure from randomness. **d**) Small-worldness for Sessions 1 and 2 had values greater than 1.0 at all threshold values ≥0.05. None of the network properties varied significantly between the two sessions at any threshold. Error bars in all graphs are ±1 standard errors of the mean. **e**) The correlation between modularity and visual short-term memory capacity varied as a function of threshold but showed comparable trends across sessions and thresholds.
(TIFF)

Figure S2 The effect of connection density (i.e., cost) threshold level on the correlation between network modularity and VSTM capacity. Connection density reflects the proportion of connections included in the network from all possible connections among the 34 nodes.
(TIFF)

Figure S3 Variation of Information (VOI) for the real and random graphs for Session 1 and Session 2, calculated individually for each subject and then averaged. Alpha is the proportion of connections between nodes that are randomly reassigned. Increased VOI reflects the loss of information between in perturbed network compared to the unperturbed network, with larger changes reflecting greater instability. Dashed line indicates the value VOI would have if 20% of the nodes were randomly assigned to new modules relative to their assignment in the original network. This reassignment was 0.15 for real networks but only 0.02 for the random networks. Note that the random networks show an immediate increase in VOI, reflecting the instability of their modularity estimates.
(TIFF)

Text S1 **Description of supplementary network thresholding analyses.**
(DOC)
