# Supplementary figures and images for: Functional Brain Network Modularity Captures Inter- and Intra-Individual Variation in Working Memory Capacity
Source: PLoS One. 2012 Jan 20;7(1):e30468. doi: 10.1371/journal.pone.0030468 (PMC3262818; doi:10.1371/journal.pone.0030468)

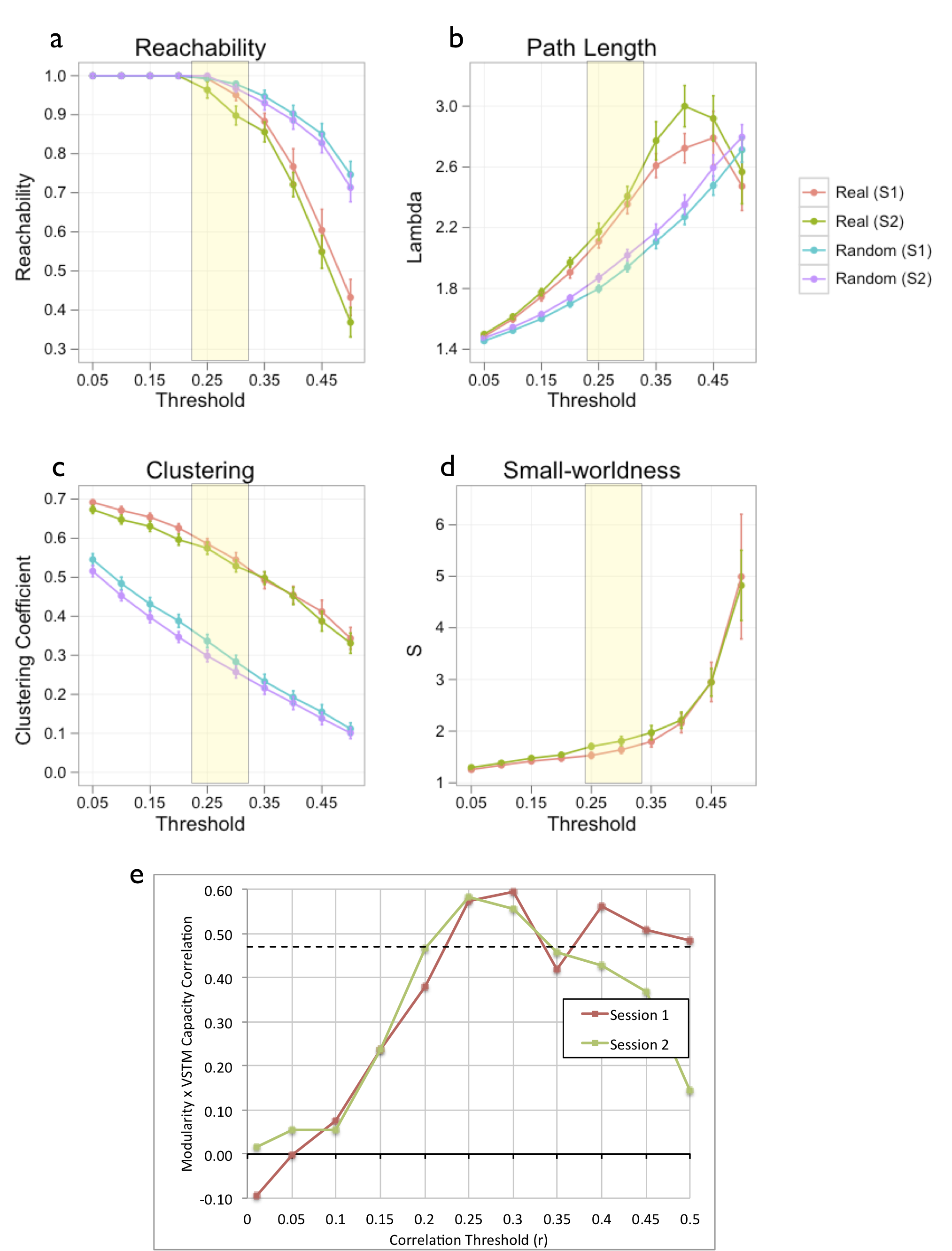

Supplement: Figure S1 — Graphs demonstrating the divergence of the real graphs from random graphs as a function of matrix thresholds for different measures of network organization. The individual networks obtained in Session 1 and Session 2 were compared to random networks constructed with the same number of nodes, connections and connection density. a) Reachability did not differ significantly from 100% when thresholds correlations were set below r = 0.30 (maximum t(16) = −1.5, p = .16), indicating that these thresholds preserve full connectedness of the network in most individual networks. b) Characteristic path length, and c) clustering coefficient were significantly different from the random graphs across all threshold correlations (r), where 0.05≤r≤0.50. c) Small-worldness (S), calculated as a ratio (Clusteringreal/Clusteringrandom)/(Path Lengthreal/Path Lengthrandom), such that values different from 1.0 reflect a departure from randomness. d) Small-worldness for Sessions 1 and 2 had values greater than 1.0 at all threshold values ≥0.05. None of the network properties varied significantly between the two sessions at any threshold. Error bars in all graphs are ±1 standard errors of the mean. e) The correlation between modularity and visual short-term memory capacity varied as a function of threshold but showed comparable trends across sessions and thresholds. (TIFF) [file pone.0030468.s001.tiff]

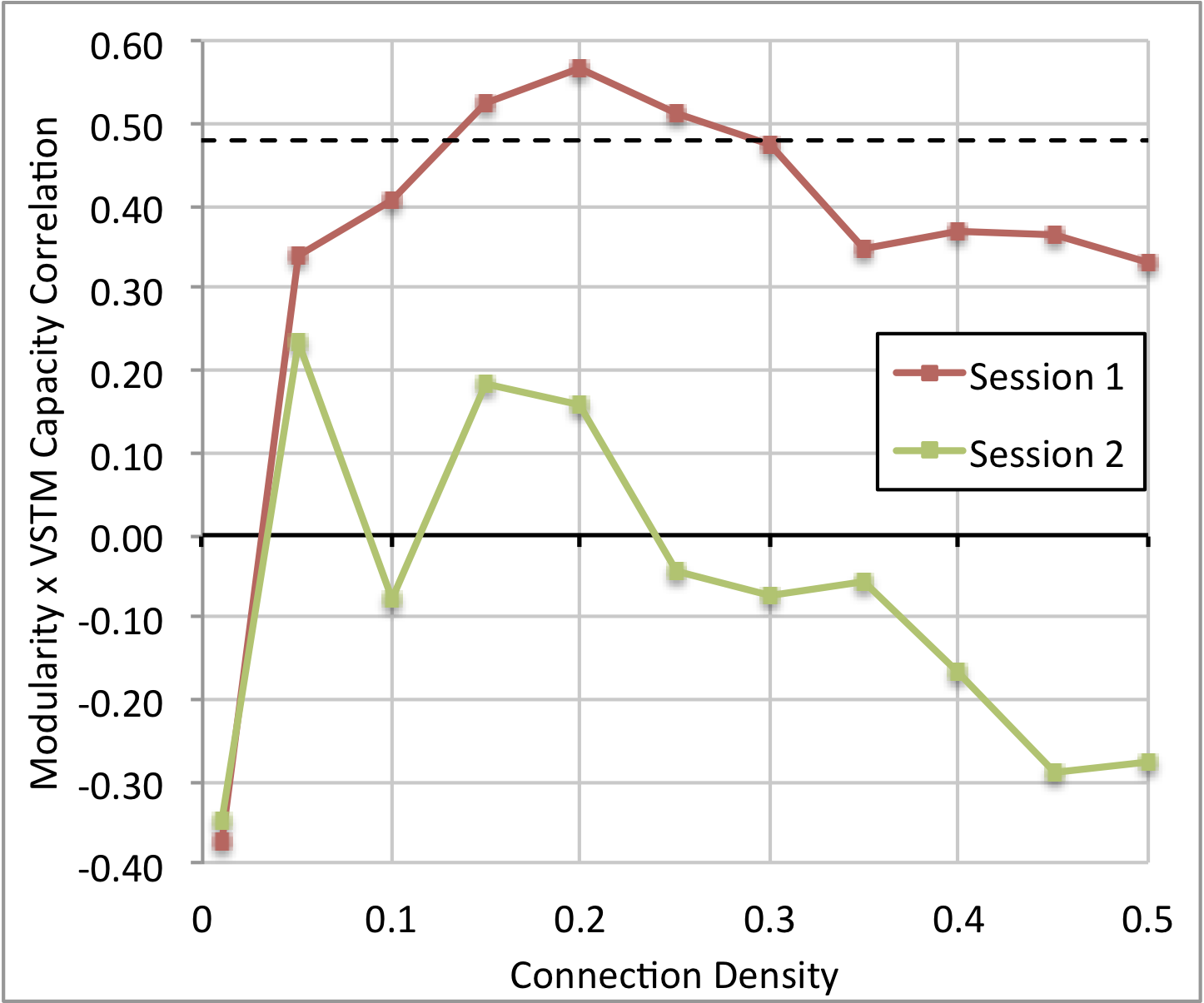

Supplement: Figure S2 — The effect of connection density (i.e., cost) threshold level on the correlation between network modularity and VSTM capacity. Connection density reflects the proportion of connections included in the network from all possible connections among the 34 nodes. (TIFF) [file pone.0030468.s002.tiff]

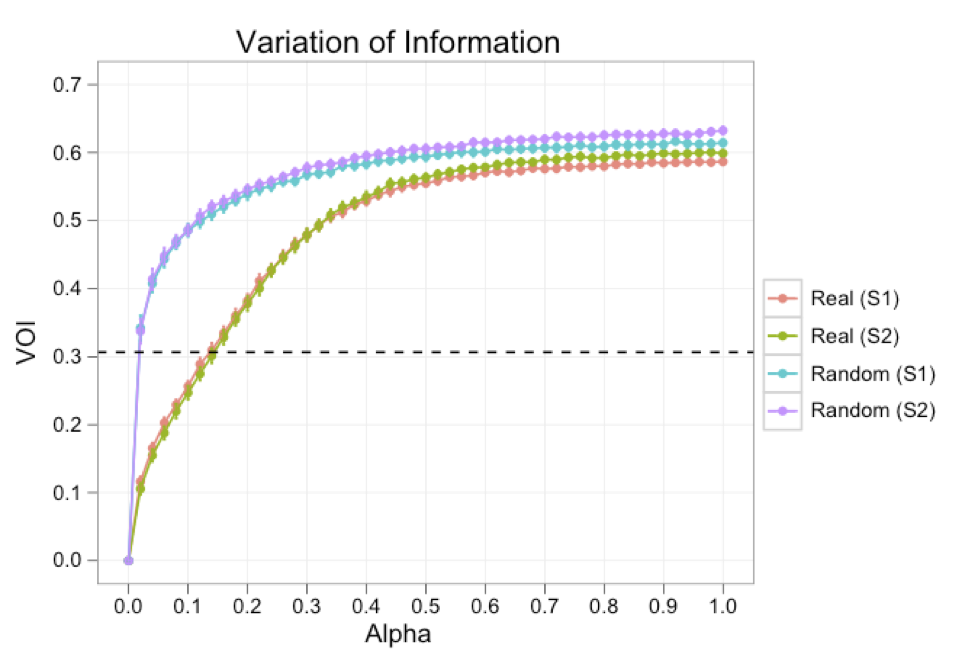

Supplement: Figure S3 — Variation of Information (VOI) for the real and random graphs for Session 1 and Session 2, calculated individually for each subject and then averaged. Alpha is the proportion of connections between nodes that are randomly reassigned. Increased VOI reflects the loss of information between in perturbed network compared to the unperturbed network, with larger changes reflecting greater instability. Dashed line indicates the value VOI would have if 20% of the nodes were randomly assigned to new modules relative to their assignment in the original network. This reassignment was 0.15 for real networks but only 0.02 for the random networks. Note that the random networks show an immediate increase in VOI, reflecting the instability of their modularity estimates. (TIFF) [file pone.0030468.s003.tiff]
