# Supplementary material for: Functional Brain Network Modularity Captures Inter- and Intra-Individual Variation in Working Memory Capacity
Source: PLoS One. 2012 Jan 20;7(1):e30468. doi: 10.1371/journal.pone.0030468 (PMC3262818; doi:10.1371/journal.pone.0030468)
Supplement: Text S1 — Description of supplementary network thresholding analyses. (DOC) [file pone.0030468.s004.doc]

Supplementary Analysis:

There are several methods for thresholding networks (e.g., [1,2]). Different approaches are based on different assumptions about the characteristics of the network. One approach is to threshold the network based on connection strength (r), typically operationalized as a correlation. When a threshold is based on a correlation the number of connections included in the network for each participant may vary as a function of the threshold and the connection strengths that exist in the network. For example, if a threshold is set at r = 0.2, some individuals may have numerous connections above 0.2, while another may have very few.

In addition to thresholding the connection matrix based on correlation strength, we also created the connection matrices using connection density (or cost). Connection density is a proportion of the number of connections among nodes over all possible connections, with the inclusion of connections based on the ranking of connection strength (correlation) between all possible node pairs. The advantage of using connection density is that it holds the number of connections equal across participant’s networks. In the current 34x34 matrix, there are 561 possible connections, so if the strongest 10% of node pairs are included, all subjects will have a binarized matrix with 56 connections. As with the correlation thresholding approach, the node pairs that are included may differ from individual to individual.

**Supplementary References**

1 Achard S, Salvador R, Whitcher B, Suckling J, Bullmore E (2006) A resilient, low-frequency, small-world human brain functional network with highly connected association cortical hubs. J Neurosci 26: 63-72.

2 van den Heuvel MP, Stam CJ, Kahn RS, Hulshoff Pol HE (2009) Efficiency of functional brain networks and intellectual performance. J Neurosci 29: 7619-7624.
